# Human herpesvirus 8 infection is associated with prostate cancer among *IFNL4-ΔG* carriers

**DOI:** 10.1038/s41391-022-00546-1

**Published:** 2022-04-25

**Authors:** Frank J. Jenkins, Tsion Z. Minas, Wei Tang, Tiffany H. Dorsey, Stefan Ambs

**Affiliations:** 1 Departments of Pathology and Infectious Diseases and Microbiology, UPMC Hillman Cancer Center, University of Pittsburgh, Pittsburgh, PA USA; 2 Laboratory of Human Carcinogenesis, Center for Cancer Research, National Cancer Institute, National Institutes of Health, Bethesda, MD USA

**Keywords:** Prostate cancer, Predictive markers

## Abstract

**Background:**

The dinucleotide germline variant, *rs368234815-ΔG*, in the *IFNL4* gene (*IFNL4-ΔG)* has been associated with prostate cancer among men at increased risk of sexually transmitted infections and reported to impair viral clearance. Human herpesvirus 8 (HHV-8) seropositivity has been associated with prostate cancer in Tobago.

**Methods:**

We examined whether the association of HHV-8 with prostate cancer is *IFNL4-ΔG*-dependent among 728 *IFNL4-ΔG*-genotyped cases and 813 genotyped population-based controls from the NCI-Maryland Prostate Cancer Case-Control study. Associations between HHV-8 and prostate cancer were assessed in multivariable unconditional logistic regression models. We calculated adjusted odds ratios (OR) and stratified the analysis into men harboring the *IFNL4-ΔG*-variant and non-carriers (ΔG/ΔG or ΔG/TT vs. TT/TT).

**Results:**

HHV-8 seropositivity was higher in cases than controls (11% vs. 6%) and this association was restricted to carriers of the ΔG allele (OR 2.19: 95% CI:1.38–3.48) in both African American (OR 1.96; 95% CI:1.08–3.56) and European American men (OR 2.59; 95% CI:1.20–5.56).

**Conclusions:**

HHV-8 seropositivity is associated with increased odds of prostate cancer in men harboring the *IFNL4* rs368234815-ΔG variant. This study describes HHV-8 infection as a candidate prostate cancer risk factor in men with the *IFNL4-ΔG* genotype and supports the hypothesis that *IFNL4-ΔG* is a susceptibility factor that contributes to prostate cancer.

## Introduction

Prostate cancer is the most common cancer among men in the United States and is second to lung cancer as a cause of cancer death [1]. The occurrence of prostate cancer is not uniform among the different US population groups. African American (AA) men experience a higher incidence and mortality than European American (EA) and Asian men [2, 3]. Despite several decades of research, the reason behind the excessive prostate cancer burden among men of African descent remains incompletely understood but we have learned that West African genetic ancestry is one underlying risk factors [4–6]. Other causes for the disease include age and family history of prostate cancer [7–10]. Modifiable risk factors such as diet and lifestyle may account for the many prostate cancers globally [11], with supporting evidence from migration studies that the environment modulates prostate cancer risk [12, 13].

Chronic inflammation is a proposed risk factor for prostate cancer and associates with aggressive disease [14, 15]. Inflammation may also contribute to prostate cancer disparities [16, 17]. While environmental exposures, such as infections, can promote inflammation, ancestral factors may additionally influence inflammatory processes and the response to infections [18, 19]. Sexually transmitted infections (STIs) including common viral infections are candidates for establishing chronic inflammation in the prostate by either repeated exposures to the infectious agent or establishment of a persistent infection [20].

Human herpesviruses are prime contenders for STIs causing chronic inflammation. Herpesviruses establish life-long latent infections and periodically reactivate resulting in viral replication and, depending on the virus, clinical disease. Several studies have failed to find associations between prostate cancer risk and infections with several herpesviruses, including herpes simplex virus, Epstein-Barr virus, and cytomegalovirus [21–24]. We, however, found that men from Tobago with prostate cancer were significantly more likely to be human herpesvirus 8 (HHV-8) seropositive than age matched controls (OR 2.24; 95% CI 1.29–3.90) [25]. Other studies including prospective US cohorts have failed to find any association between HHV-8 seropositivity and prostate cancer risk [26, 27]. HHV-8 is sexually transmitted and the causative agent of Kaposi’s sarcoma and pleural effusion lymphomas [28]. Notably, HHV-8 can establish latent infections in the prostate glandular epithelium and the infection was found associated with an increased macrophage infiltrate [29, 30]. Thus, HHV-8 may serve as one of the infectious agents that others have hypothesized as responsible for establishing chronic infection, leading to increased inflammation in the prostate and an elevated risk of prostate cancer.

Interferon lambda 4 (*IFNL4*) is a type III interferon discovered in 2013 which can serve as a negative regulator of IFN responses [31]. Recently, a germline dinucleotide polymorphism, rs368234815 (ΔG and TT alleles) in the interferon lambda 4 gene has been reported to modify the association of sexual history with prostate cancer [32, 33]. Upon induction, both ΔG and TT variants produce *IFNL4* mRNA. However, IFN-λ4 protein can only be produced in the presence of the ΔG allele because the TT allele encodes a premature stop codon for translation into protein [31]. The *IFNL4-ΔG* variant, which results in the expression of IFN-λ4 protein, is strongly linked to an inability to clear certain chronic viral infections such as hepatitis C virus [31, 34, 35] and cytomegalovirus [36, 37]. The *IFNL4-ΔG* variant is also more prominent among African (78%) than European (30%) or Asian men (0–10%) [31]. Similarly, we find that the *IFNL4-ΔG* variant has a 61.7% allele frequency among AA men and 33.7% allele frequency among EA men in the NCI-Maryland Prostate Cancer Case-Control study. Thus, the presence of this polymorphism may predispose to prostate cancer in the context of a pro-inflammatory infection but may affect AA men more so than EA men because of its high germline prevalence among AA men.

To examine whether an interaction between HHV8 and *IFNL4-ΔG* may increase the risk of prostate cancer, we investigated the combined effect of an HHV-8 infection history and *IFNL4-ΔG* on prostate cancer in the NCI-Maryland Prostate Cancer Case-Control Study.

## Materials and methods

### NCI-Maryland prostate cancer case-control study

This case-control study has been previously described [33, 38]. A key purpose of this study was to investigate the role of environmental and genetic factors in prostate cancer development among AA men. Prior to interview, all participants signed informed consent for participation. All study forms and procedures were approved by the NCI (protocol # 05-C-N021) and the University of Maryland (protocol #0298229) Institutional Review Boards. Cases were recruited at the Baltimore Veterans Affairs Medical Center and the University of Maryland Medical Center. The use of data and samples from this study was approved as an exempt study by the University of Pittsburgh Institutional Review Board (PRO18060266). Eligibility criteria included the following: diagnosis with prostate cancer within two years prior to enrollment, residence in Maryland or adjacent counties in Pennsylvania, Delaware, Virginia, or District of Columbia, 40–90 years old at the time of enrollment, born in the United States, either AA or EA by self-report, can be interviewed in English, had a working home phone number, physically and mentally fit to be interviewed, not severely ill, and not residing in an institution such as prison, nursing home, or shelter. A total of 976 cases (489 AA and 487 EA men) were recruited into the study between 2005 and 2015. Controls were identified through the Maryland Department of Motor Vehicle Administration database and were frequency-matched to cases on age and race. The controls also had the same eligibility criteria as cases with the exception that they could not have a personal history of cancer (other than non-melanoma skin cancer), radiation therapy, or chemotherapy. A total of 1034 population controls were recruited (486 AA and 548 EA men). At the time of enrollment, both cases and controls were administered a survey by a trained interviewer and a blood sample or mouthwash rinse/buccal cells was collected. The survey asked about their demographics, tobacco use, nutrition, medical history, family history of cancer, prostatitis, or benign prostatic hypertrophy, occupational history, socioeconomic status, anthropometry, and sexual history. For the current study, a total of 1729 participants (851 cases, 878 controls) were included in the analysis, based on availability of sera and survey data, and the exclusion of HHV8 test results because of quality control failure (1.2%). Because *IFNL4-ΔG* genotyping data was not obtained for 188 participants who did not provide germline DNA, only 1541 participants (728 cases and 813 controls) were included in the combined assessment of HHV-8 serology and the *IFNL4-ΔG* genotype in prostate cancer development. The 188 participants without genotyping data had similar characteristics as the genotyped participants. In the study, we report prostate-specific antigen (PSA) levels for the control population. PSA measurements in the stored sera samples were performed years after recruitment of these controls when recruitment into the study was completed. We did not use the PSA measurements for selection of controls.

### HHV-8 serology

HHV-8 serology was obtained for 1751 participants, but 22 results were excluded from further analysis because of quality control failure (1.2%, with high background making seropositivity determination impossible). HHV-8 serology was determined using a well-defined immunofluorescence assay (IFA) [39–41]. The inter-assay reliability with the HHV-8 IFA is 93.4%. All serum samples were tested at least twice in a blinded fashion and assessed microscopically by the same reader. The cutoff for a positive serologic result was 1:50.

### Genotyping of IFNL4 rs368234815

This assay has been previously described [33]. Genomic DNA was isolated from buffy coats, when available, using the DNeasy Blood &Tissue Kit (Qiagen, Germantown, MD). For a subset of men (100 cases and 112 controls), genomic DNA was obtained from a mouthwash sample using the Gentra Puregene Buccal Cell Kit (Qiagen). Genotyping of rs368234815 was done with a custom-designed assay purchased from ThermoFisher (Waltham, MA) and was performed using ABI 7900 (Applied Biosystems, (Waltham, MA) according to the standard protocol. Genotype concordance among duplicates was 97%. There was no deviation from the Hardy–Weinberg equilibrium among controls. Genotyping success rate was 98.6%. The genotyping failure rate was similarly low for DNA extracted from blood and mouthwash samples (1.4%).

### Prostate cancer classification

To define high grade disease, Gleason grading system was used to stratify prostate cancer into “low-grade” (Gleason score ≤ 7) or “high-grade” (Gleason score > 7). Tumor stage at diagnosis was obtained from medical records and stage 1 or 2 disease was defined as localized and stage 3 or 4 as advanced prostate cancer. Cases with a combination of low-grade tumors and localized disease were defined as “nonaggressive” prostate cancer, while cases with either high-grade tumors or advanced disease were defined as having “aggressive” prostate cancer.

### Statistics

All statistical analyses were performed using the Stata/SE 16.0 statistical software package (Stata Corp, College Station, TX). All statistical tests were two-sided and *P* < 0.05 was considered statistically significant. We evaluated whether HHV-8 is associated with prostate cancer and then stratified by race/ethnicity (AA vs. EA) and *IFNL4* rs368234815 (ΔG/ΔG or ΔG/TT vs. TT/TT), or by aggressive vs. nonaggressive disease. To assess the association of HHV-8 with prostate cancer, unconditional logistic regression was used to compute odds ratios (OR) and 95% confidence intervals (CI). We adjusted for the following potential confounding factors: age at enrollment, body mass index at enrollment (BMI, kg m^−2^), education (high school or less, some college, college, professional school), family history of prostate cancer (first degree relatives, yes/no), smoking history (never, former, current), condom use (usually use, yes/no), aspirin use (regular user, yes/no), and *IFNL4* rs368234815 (ΔG/ΔG or ΔG/TT vs. TT/TT) or race/ethnicity as applicable.

## Results

### Clinical and demographic characteristics of participants

Characteristics of the study participants are shown in Table 1. Cases and controls had similar age distributions (Wilcoxon rank-sum test, *P* = 0.15). Cases had slightly lower BMI than controls (Wilcoxon rank-sum test, *P* = 0.028). More cases, compared to controls, had a first-degree family history of prostate cancer (10% vs. 7%, *P* = 0.019, Chi-square test), whereas fewer cases than controls had a college or graduate-level degree (34% vs. 51%, *P* < 0.001, Chi-square test). Additionally, more cases were current smokers than controls (24% vs. 14%, *P* < 0.001, Chi-square test). Among the 851 cases, 121 men (14%) had advanced disease (stage 3 or 4 prostate cancer). High Gleason score (>7) was reported in 145 patients (17%). Both AA and EA men had similar Gleason score distribution, but AA men tended to have higher blood PSA levels at diagnosis. A somewhat greater proportion of EA men (15%), compared with AA men (13%), presented with an advanced stage disease in this case-control study.

**Table 1:** Characteristics of study participants.

	Controls			Cases^a^		
	All (*n* = 878)	AA^b^ (*n* = 406)	EA^c^ (*n* = 472)	All (*n* = 851)	AA (*n* = 412)	EA (*n* = 439)
*Demographic factors*						
Age^d^						
Median (IQR^e^) in years	64 (12)	64 (10)	66 (13)	64 (11)	63 (10)	65 (11)
*BMI*						
Mean (SD^f^) in kg/m2	28 (6.5)	29.6 (5.4)	27.9 (4.9)	27.4 (5.6)	27.9 (5.2)	28.0 (4.3)
*Education*, *N* (%)						
High school or less	209 (24)	119 (29)	90 (19)	313 (37)	196 (48)	117 (27)
Some college	220 (25)	119 (29)	101 (21)	250 (29)	136 (33)	114 (26)
College	226 (26)	87 (21)	139 (29)	159 (19)	53 (13)	106 (24)
Graduate	222 (25)	80 (20)	142 (30)	128 (15)	26 (6)	102 (23)
Did not provide	1 (<1)	1 (<1)	–	1 (<1)	1 (<1)	–
*Annual household income*, *N* (%)						
Less than $10,000	25 (3)	19 (5)	6 (1)	79 (9)	63 (15)	16 (4)
$10,000–$29,999	115 (13)	72 (18)	43 (9)	226 (27)	143 (35)	83 (19)
$30,000–$59,999	200 (23)	89 (22)	111 (24)	188 (22)	95 (23)	93 (21)
$60,000–$90,000	205 (23)	96 (24)	109 (23)	130 (15)	41 (10)	89 (20)
>$90,000	266 (30)	92 (23)	174 (37)	161 (19)	31 (8)	130 (30)
Don’t know/Refused	67 (8)	38 (9)	29 (6)	67 (8)	39 (9)	28 (6)
*Health factors*						
Family history of prostate cancer^g^, *N (%)*						
No	817 (93)	381 (94)	436 (92)	765 (90)	376 (91)	389 (89)
Yes	61 (7)	25 (6)	36 (8)	86 (10)	36 (9)	50 (11)
Smoking status^h^, *N* (%)						
Never	349 (40)	158 (39)	191 (40)	291 (34)	119 (29)	172 (39)
Former	398 (45)	167 (41)	231 (49)	355 (42)	155 (38)	200 (46)
Current	122 (14)	78 (19)	44 (9)	200 (24)	135 (33)	65 (15)
Did not provide	9 (1)	3 (1)	6 (1)	5 (<1)	3 (1)	2 (<1)
Diabetes, *N* (%)						
No	677 (77)	286 (70)	391 (83)	671 (79)	297 (72)	374 (85)
Yes	201 (23)	120 (30)	81 (17)	180 (21)	115 (28)	65 (15)
Regular aspirin use, *N* (%)						
No	377 (43)	197 (49)	180 (38)	433 (51)	238 (58)	195 (44)
Yes	501 (57)	209 (51)	292 (62)	418 (49)	174 (42)	244 (56)
Gleason grade group, *N* (%)						
Gleason grade group 1 (6 or less)	NA	NA	NA	363 (43)	167 (41)	196 (45)
Gleason grade group 2 (3 + 4)	NA	NA	NA	233 (27)	118 (29)	115 (26)
Gleason grade group 3 (4 + 3)	NA	NA	NA	108 (13)	57 (14)	51 (12)
Gleason grade group 4 (8)	NA	NA	NA	83 (10)	41 (10)	42 (9)
Gleason grade group 5 (9 or 10)	NA	NA	NA	62 (7)	28 (7)	34 (8)
Not available	NA	NA	NA	2 (<1)	1 (<1)	1 (<1)
Stage^i^, *N* (%)						
I	NA	NA	NA	163 (19)	63 (15)	100 (23)
II	NA	NA	NA	567 (67)	297 (72)	270 (62)
III	NA	NA	NA	69 (8)	24 (6)	45 (10)
IV	NA	NA	NA	52 (6)	28 (7)	24 (5)
PSA^j^						
Median (IQR) in ng/ml	0.4 (0.6)	0.4 (0.6)	0.4 (0.6)	6.3 (5.6)	6.8 (7.6)	6.0 (4.7)
IFNL4 rs368234815 genotype, *N* (%)						
IFNL4 TT/TT	255 (31)	59 (15)	196 (46)	225 (31)	59 (16)	166 (45)
IFNL4 ΔG/TT	361 (44)	185 (48)	176 (41)	317 (44)	157 (44)	160 (43)
IFNL4 ΔG/ΔG	197 (24)	139 (36)	58 (13)	186 (26)	142 (40)	44 (12)
HHV-8 seroprevalence, *N* (%)						
Positive	57 (6)	30 (7)	27 (6)	92 (11)	52 (13)	40 (9)
Negative	821 (94)	376 (93)	445 (94)	759 (89)	360 (87)	399 (91)

^a^Cases recruited within 2 years after disease diagnosis with a median interval between diagnosis and enrollment of 5.1 months.

^b^AA: African-American.

^c^EA: European-American.

^d^Age at study interview.

^e^IQR: Interquartile range.

^f^SD: Standard deviation.

^g^First-degree relative with prostate cancer.

^h^Smoking status describes cigarette smoking.

^i^Pathologically confirmed using American Joint Committee on Cancer (AJCC) 7th Edition.

^j^PSA: Prostate specific antigen.

### HHV-8 and prostate cancer

The HHV-8 seroprevalence among study participants was 9.4%, with an increased seropositivity among AA men (11.1%) compared to EA men (7.9%; unadjusted OR 1.40; 95% CI: 1.001–1.97, AA vs. EA men). To determine if HHV-8 seroprevalence is associated with prostate cancer among study participants, univariate and multivariable analyses were performed with and without stratification by self-reported race/ethnicity (Table 2). As shown in Table 1, HHV-8 seroprevalence was higher among men with prostate cancer (11%) when compared to the control group (6%; unadjusted OR 1.75; 95% CI: 1.23–2.46) and remained significantly elevated among cases in the confounder-adjusted logistic regression analysis that included *IFNL4* rs368234815 (ΔG/ΔG or ΔG/TT vs. TT/TT) as covariate (adjusted OR 1.76; 95% CI: 1.20–2.59; Table 2). After stratification by race/ethnicity, the association of HHV-8 seropositivity with prostate cancer remained significant only among AA men (adjusted OR 1.91; 95% CI: 1.11–3.27). However, among EA men, a similar trend was observed (adjusted OR 1.57: 95% CI: 0.90–2.75).

**Table 2:** Association between HHV8 and prostate cancer stratified by self-reported race/ethnicity.

	Total					African-American					European-American				
	Control *N* (%)	Case *N* (%)	Univariable OR (95% CI)	Multivariable^a^ OR (95% CI)	*P-*value	Control *N* (%)	Case *N* (%)	Univariable OR (95% CI)	Multivariable^b^ OR (95% CI)	*P-*value	Control *N* (%)	Case *N* (%)	Univariable OR (95% CI)	Multivariable^b^ OR (95% CI)	*P-*value
HHV8															
No	821 (94)	759 (89)	Ref.	Ref.		376 (93)	360 (97)	Ref.	Ref.		445 (94)	399 (91)	Ref.	Ref.	
Yes	57 (6)	92 (11)	1.75 (1.23, 2.46)	**1.76 (1.20, 2.59)**	**0.004**	30 (7)	52 (13)	1.81 (1.13, 2.90)	**1.91 (1.11, 3.27)**	**0.019**	27 (6)	40 (9)	1.65 (1.00, 2.74)	1.57 (0.90, 2.75)	0.113

Bolded data indicate significant associations in the multivariable logistic regression analysis.

OR denotes Odds Ratio; 95% CI denotes 95% confidence interval.

^a^Unconditional logistic regression adjusted for body mass index at study enrollment (BMI, kg/m^2^), age at study entry, education (high school or less, some college, college, professional school), family history of prostate cancer (first degree relatives, yes/no), smoking history (never, former, current), condom use (usually use, yes/no), aspirin use (regular user, yes/no), IFNL4 rs368234815 genotype (ΔG/ΔG or ΔG/TT vs. TT/TT), and race.

^b^Unconditional logistic regression adjusted for body mass index at study enrollment (BMI, kg/m^2^), age at study entry, education (high school or less, some college, college, professional school), family history of prostate cancer (first degree relatives, yes/no), smoking history (never, former, current), condom use (usually use, yes/no), aspirin use (regular user, yes/no), and IFNL4 rs368234815 genotype (ΔG/ΔG or ΔG/TT vs. TT/TT).

### Influence of *IFNL4* rs368234815 genotype on the association between HHV-8 and prostate cancer

*IFNL4* rs368234815 genotype frequencies are shown in Table 1. There is a significant overrepresentation of homozygote *IFNL4-ΔG* carriers among AA men, yielding a *ΔG* allele frequency of 61.7% in AA vs. 33.7% in EA men, consistent with the literature [31]. To determine if the *IFNL4* rs368234815 genotype modifies the association between HHV-8 and prostate cancer, we stratified the participants into those carrying a ΔG allele (ΔG/TT or ΔG/ΔG) vs. those without the ΔG allele (TT/TT). The association of HHV-8 seropositivity with prostate cancer was restricted to carriers of the ΔG allele (adjusted OR 2.19: 95% CI: 1.38–3.48) in the multivariable analysis that did not stratify by race/ethnicity (Table 3). Carriers of the TT genotype were not at increased odds of prostate cancer due to HHV-8 seropositivity (adjusted OR 1.03: 95% CI: 0.51–2.11). Further stratification by race/ethnicity showed that HHV-8 was significantly associated with prostate cancer only among carriers of the ΔG allele in both EA (OR 2.59; 95% CI: 1.20–5.56) and AA men (OR 1.96; 95% CI:1.08–3.56). Although the OR point estimate for the association of HHV-8 with prostate cancer in men with *IFNL4-ΔG* was higher in EA than AA men (2.59 vs. 1.96), the 95% CIs largely overlapped, suggesting comparable risks. However, among AA men, HHV-8 showed a trend towards an association among *IFNL4* TT carriers, albeit not statistically significant. The association of the *IFNL4-ΔG* genotype with prostate cancer risk among men who were HHV-8 seropositive suggests the existence of a gene–environment interaction.

**Table 3:** Association between HHV-8 and prostate cancer after stratification by *IFNL4* rs368234815 genotype.

**Total**										
	**IFNL4 ΔG/TT or ΔG/ΔG**					**IFNL4 TT/TT**				
	**Control** ***N*** **(%)**	**Case** ***N*** **(%)**	**Univariable OR (95% CI)**	**Multivariable**^**a**^ **OR (95% CI)**	***P-*value**	**Control** ***N*(%)**	**Case** ***N*** **(%)**	**Univariable OR (95% CI)**	**Multivariable**^**a**^ **OR (95% CI)**	***P-*value**
**HHV-8**										
No	527 (94)	441 (88)	Ref.	Ref.		237 (93)	205 (91)	Ref.	Ref.	
Yes	31 (6)	62 (12)	2.39 (1.53, 3.75)	**2.19 (1.38, 3.48)**	**0.001**	18 (7)	20 (9)	1.28 (0.66, 2.49)	1.03 (0.51, 2.11)	0.922
**European-American**										
	**IFNL4 ΔG/TT or ΔG/ΔG**					**IFNL4 TT/TT**				
	**Control** ***N*** **(%)**	**Case** ***N*** **(%)**	**Univariable OR (95% CI)**	**Multivariable**^**b**^ **OR (95% CI)**	***P-*value**	**Control** ***N*** **(%)**	**Case** ***N*** **(%)**	**Univariable OR (95% CI)**	**Multivariable**^**b**^ **OR (95% CI)**	***P-*value**
**HHV-8**										
No	223 (95)	180 (88)	Ref.	Ref.		182 (93)	157 (95)	Ref.	Ref.	
Yes	11(5)	24 (12)	2.70 (1.29, 5.67)	**2.59 (1.20, 5.56)**	**0.015**	14 (7)	9 (5)	0.75 (0.31, 1.77)	0.76 (0.30, 1.88)	0.547
**African-American**										
	**IFNL4 ΔG/TT or ΔG/ΔG**					**IFNL4 TT/TT**				
	**Control** ***N*** **(%)**	**Case** ***N*** **(%)**	**Univariable OR (95% CI)**	**Multivariable**^**b**^ **OR (95% CI)**	***P-*value**	**Control** ***N*** **(%)**	**Case** ***N*** **(%)**	**Univariable OR (95% CI)**	**Multivariable**^**b**^ **OR (95% CI)**	***P-*value**
**HHV-8**										
No	304 (94)	261 (87)	Ref.	Ref.		55 (93)	48 (81)	Ref.	Ref.	
Yes	20 (6)	38 (13)	2.21 (1.26, 3.90)	**1.96 (1.08, 3.56)**	**0.027**	4 (7)	11 (19)	3.15 (0.94, 10.55)	1.45 (0.38, 5.52)	0.579

Bolded data indicate significant associations in the multivariable logistic regression analysis.

OR denotes odds ratio; 95% CI denotes 95% confidence interval.

^a^Unconditional logistic regression adjusted for body mass index at study enrollment (BMI, kg/m^2^), age at study entry, education (high school or less, some college, college, professional school), family history of prostate cancer (first degree relatives, yes/no), smoking history (never, former, current), condom use (usually use, yes/no), aspirin use (regular user, yes/no), and race.

^b^Unconditional logistic regression adjusted for body mass index at study enrollment (BMI, kg/m^2^), age at study entry, education (high school or less, some college, college, professional school), family history of prostate cancer (first degree relatives, yes/no), smoking history (never, former, current), condom use (usually use, yes/no), and aspirin use (regular user, yes/no).

### HHV-8 and risk of aggressive prostate cancer

To determine if the association between HHV-8 and prostate cancer risk differed between aggressive vs. non-aggressive prostate cancer, we looked at nonaggressive and aggressive prostate cancer as separate outcomes. The association of HHV-8 with aggressive prostate cancer was slightly stronger (OR 1.77; 95% CI: 1.06–2.96) than the association of HHV-8 with non-aggressive prostate cancer (OR 1.51; 95% CI: 1.02–2.22), yet the confidence intervals largely overlapped (Supplementary Table 1). In the analysis stratified by race/ethnicity, we found a significant association between HHV-8 and the risk for aggressive prostate cancer among AA men (OR 2.69; 95% CI: 1.27–5.68), but not among EA men (OR 1.38; 95% CI: 0.59–3.23), in the multivariable analysis (Table 4). The association of HHV-8 with non-aggressive prostate cancer was weaker and did not reach statistical significance in either group of men. In contrast, HHV-8 was associated with both aggressive (OR 2.62; 95% CI: 1.36–5.06) and non-aggressive prostate cancer (OR 2.06; 95% CI: 1.25–3.38) among men carrying at least one ΔG allele (Table 5), again indicating that HHV-8 is a prostate cancer risk factor if men harbor the *IFNL4-ΔG* germline variant—independent of race/ethnicity.

**Table 4:** Association of HHV-8 with aggressive vs. non-aggressive prostate cancer stratified by self-reported race/ethnicity.

	African-American					European-American				
	Control *N* (%)	Aggressive Case *N* (%)	Non-aggressive Case *N* (%)	Aggressive Case Multivariable^a^ OR (95% CI)	Non-Aggressive Case Multivariable^a^ OR (95% CI)	Control *N (%*)	Aggressive Case *N* (%)	Non-aggressive Case *N* (%)	Aggressive Case Multivariable^a^ OR (95% CI)	Non-Aggressive Case Multivariable^a^ OR (95% CI)
HHV-8										
No	376 (93)	83 (84)	277 (89)	Ref.	Ref.	445 (94)	106 (92)	293 (90)	Ref.	Ref.
Yes	30 (7)	16 (16)	36 (12)	**2.69 (1.27, 5.68)**	1.59 (0.89, 2.84)	27 (6)	9 (8)	31 (10)	1.38 (0.59, 3.23)	1.75 (0.95, 3.21)

Bolded data indicate significant associations in the multivariable logistic regression analysis.

OR denotes Odds Ratio; 95% CI denotes 95% confidence interval.

^a^Unconditional logistic regression adjusted for body mass index at study enrollment (BMI, kg/m^2^), age at study entry, education (high school or less, some college, college, professional school), family history of prostate cancer (first degree relatives, yes/no), smoking history (never, former, current), condom use (usually use, yes/no), aspirin use (regular user, yes/no), and IFNL4 rs368234815 genotype (ΔG/ΔG or ΔG/TT vs. TT/TT).

**Table 5:** Association of HHV-8 with aggressive vs. non-aggressive prostate cancer stratified by *IFNL4* rs368234815 genotype.

	IFNL4 ΔG/TT or ΔG/ΔG					IFNL4 TT/TT				
	Control *N* (%)	Aggressive Case *N* (%)	Non-aggressive Case *N* (%)	Aggressive Case Multivariable^a^ OR (95% CI)	Non-Aggressive CaseMultivariable^a^ OR (95% CI)	Control *N* (%)	Aggressive Case *N* (%)	Non-aggressive Case *N* (%)	Aggressive Case Multivariable^a^ OR (95% CI)	Non-Aggressive Case Multivariable^a^ OR (95% CI)
HHV-8										
No	527 (94)	109 (87)	332 (88)	Ref.	Ref.	237 (93)	56 (90)	149 (91)	Ref.	Ref.
Yes	31 (6)	17 (13)	45 (12)	**2.62 (1.36, 5.06)**	**2.06 (1.25, 3.38)**	18 (7)	6 (10)	14 (9)	1.26 (0.46, 3.46)	1.00 (0.45, 2.22)

Bolded data indicate significant associations in the multivariable logistic regression analysis.

OR denotes Odds Ratio; 95% CI denotes 95% confidence interval.

^a^Unconditional logistic regression adjusted for body mass index at study enrollment (BMI, kg/m^2^), age at study entry, education (high school or less, some college, college, professional school), family history of prostate cancer (first degree relatives, yes/no), smoking history (never, former, current), condom use (usually use, yes/no), aspirin use (regular user, yes/no), and race.

## Discussion

Here, we report that HHV-8 seropositivity is associated with prostate cancer in men harboring the *IFNL4* rs368234815-ΔG germline variant, suggesting a gene–environment interaction that increases prostate cancer risk. Although our findings do not show how HHV-8 may affect prostate cancer biology, the presence of *IFNL4-ΔG* may interfere with the clearance of an HHV-8 infection, thereby conferring the risk of developing prostate cancer.

Chronic viral infections are thought to commonly establish an oncogenic proinflammatory environment. Chronic low-grade inflammation has been proposed as a driving force behind the initiation and development of prostate cancer [42]. While an infectious agent as the cause of prostate cancer has not been conclusively established, Nelson and colleagues hypothesized that host immune inflammatory responses to infections may serve as the causative process for prostate cancer [20]. Hayes et al. [43] reported an elevated risk of prostate cancer among men with STIs, yet other studies could not make this association [44].

HHV-8 establishes latent infections in the host which can lead to chronic inflammation due to the expression of a subset of proteins termed the latency proteins [28]. Thus, we hypothesized that an HHV-8 infection may increase prostate cancer risk in susceptible individuals, potentially leading to population differences in prostate cancer risk associated with HHV-8. Detection of HHV-8-specific antibodies does not indicate when the individual was infected with HHV-8 but does demonstrate that they are latently infected since all human herpesviruses establish latent infections in the host. Our previous studies investigating HHV-8 and prostate cancer in the Caribbean Island of Tobago supported this hypothesis. In Tobago, HHV-8 seroprevalence was significantly higher among Tobago men (31.5%) compared to EA men in the US (13%) or U.S. blood donors overall (4.3%) [25, 45]. Because HHV-8 establishes a chronic, latent infection in the glandular epithelium of the prostate, the virus’ presence results in an increased macrophage infiltrate [29, 30].

In the present study, we have extended our earlier studies on HHV-8 and prostate cancer from Tobago to the U.S. HHV-8 (as determined by seropositivity) was found to be significantly associated with prostate cancer in the NCI-Maryland Prostate Cancer Case Control Study. This association was most obvious among AA men. Nevertheless, the increased odds of prostate cancer were chiefly associated with the occurrence of the *IFNL4-ΔG* germline variant, and this relationship was principally independent of race/ethnicity. Noteworthy, this is the first study to demonstrate an association between HHV-8 and prostate cancer risk among AA men. In smaller studies investigating HHV-8 and prostate cancer risk in the US, we previously failed to find an association, but these studies were primarily focused on EA men [26, 27] and did not consider *IFNL4-ΔG* as an important modulator of the association between HHV-8 and prostate cancer risk. Moreover, those studies were limited in sample size and lacked sufficient power given the relatively low seroprevalence of HHV-8 in the U.S (4.3–13%) [25, 41], when compared to Tobago (31.5%) [25]. Additionally, the prevalence of *IFNL4-ΔG* is lower in EA men than men of African ancestry [31, 33].

The present study also extends the observations from an earlier study that found the *IFNL4-ΔG* genotype to be associated with prostate cancer among men of an increased risk of sexually transmitted infections [33]. Based on the findings, the authors proposed that an infectious viral agent(s), capable of establishing a chronic infection in the background of the *IFNL4-ΔG* genotype, resulted in the increased prostate cancer risk. IFN-λ4 is involved in the innate immune response and its expression is associated with an inability to clear certain viral infections such as hepatitis C virus [31, 34, 35] and cytomegalovirus [36, 37]. Thus, it is plausible that *IFNL4-ΔG* may increase prostate cancer risk by interfering with the clearance of a viral infection like HHV-8.

Our study has limitations. They include the case-control design, not allowing us to evaluate whether HHV-8 seropositivity precedes prostate cancer development. In addition, we could not evaluate the relationship of HHV-8 seropositivity with prostate cancer for all men in the NCI-Maryland study because we could not collect sera samples and germline DNA from a subset of the men, leading to the inclusion of 1541 men in the analysis out of the 2010 recruited men (76.7%). Future studies are needed that use a prospective design to establish HHV-8 as a prostate cancer risk factor among men with the *IFNL4-ΔG* genotype.

In summary, the present study provides first evidence that HHV-8 seropositivity is associated with increased odds of prostate cancer in men harboring the *IFNL4* rs368234815-ΔG germline variant, consistent with previous data from this group. The observations suggest that HHV-8 represents an infectious viral agent that establishes a chronic infection in the prostate in an *IFNL4-ΔG* genotype-dependent manner, and thereby increases prostate cancer risk. The expression of IFN-λ4 protein may prevent the host from clearing the chronic infection, as has been suggested [34], leading to prostate cancer.

## Supplementary information

Association of HHV-8 with aggressive vs. non-aggressive prostate cancer

## Data Availability

Data sharing not applicable to this article as no datasets were generated or analyzed during the current study.
