# Supplementary material for: Human herpesvirus 8 infection is associated with prostate cancer among IFNL4-ΔG carriers
Source: Prostate Cancer Prostatic Dis. 2022 Apr 25;26(2):338–46. doi: 10.1038/s41391-022-00546-1 (PMC9592685; doi:10.1038/s41391-022-00546-1)
Supplement: Supplementary file 1 — Association of HHV-8 with aggressive vs. non-aggressive prostate cancer [file 41391_2022_546_MOESM1_ESM.pdf]

Supplementary Table 1. Association of HHV-8 with aggressive vs. non-aggressive prostate cancer

	Control *N (%)*	Aggressive Case *N (%)*	Non-aggressive Case *N (%)*	Aggressive Case Multivariable[a] OR (95% CI)	Non-Aggressive Case Multivariable[a] OR (95% CI)
**HHV-8**					
No	821 (94)	189 (88)	570 (89)	Ref.	Ref.
Yes	57 (6)	25 (12)	67 (11)	**1.77 (1.06, 2.96)**	**1.51 (1.02, 2.22)**

[a]Unconditional logistic regression adjusted for body mass index at study enrollment (BMI, kg/m^2), age at study entry, education (high school or less, some college, college, professional school), family history of prostate cancer (first degree relatives, yes/no), smoking history (never, former, current), condom use (usually use, yes/no), aspirin use (regular user, yes/no), and race

OR denotes Odds Ratio; 95% CI denotes 95% confidence interval

NOTE: Bolded data indicate significant associations in the multivariable logistic regression analysis.
